# (1*S*,2*R*,6*R*,7a*S*)-1,2,6-Trihy­droxy­hexa­hydro-1*H*-pyrrolizin-3-one

**DOI:** 10.1107/S1600536812002292

**Published:** 2012-02-04

**Authors:** F. L. Oliveira, K. R. L. Freire, R. Aparicio, F. Coelho

**Affiliations:** a Laboratory of Structural Biology and Crystallography, Institute of Chemistry, University of Campinas, CP6154, CEP 13083-970, Campinas-SP, Brazil; b Laboratory of Synthesis of Natural Products and Drugs, Institute of Chemistry, University of Campinas, CP6154, CEP 13083-970, Campinas-SP, Brazil

## Abstract

In the title compound, C_7_H_11_NO_4_, prepared *via* a Morita–Baylis–Hillman adduct, the five-membered ring bearing three O atoms approximates to a twisted conformation, whereas the other ring is close to an envelope, with a C atom in the flap position. The dihedral angle between their mean planes (all atoms) is 23.11 (9)°. The new stereocenters are created in a *trans*-diaxial configuration. In the crystal, O—H⋯O and O—H⋯(O,O) hydrogen bonds link the mol­ecules, generating a three-dimensional network. A weak C—H⋯O inter­action also occurs.

## Related literature

For the utilization of this type of pyrrolizidinone as an inihibitor of glicosidase, see: D’Alanzo *et al.* (2009) ▶; Ayad *et al.* (2004 ▶) and for their huge therapeutical potential for the treatment of a number of diseases such as cancer, diabetes, and lysosomal storage disorders, see: Baumann (2007 ▶). For related literature concerning preparation of the title compound, see: Freire *et al.* (2007 ▶). Analysis of the absolute structure was also performed using likelihood methods, see: Hooft *et al.* (2008 ▶).
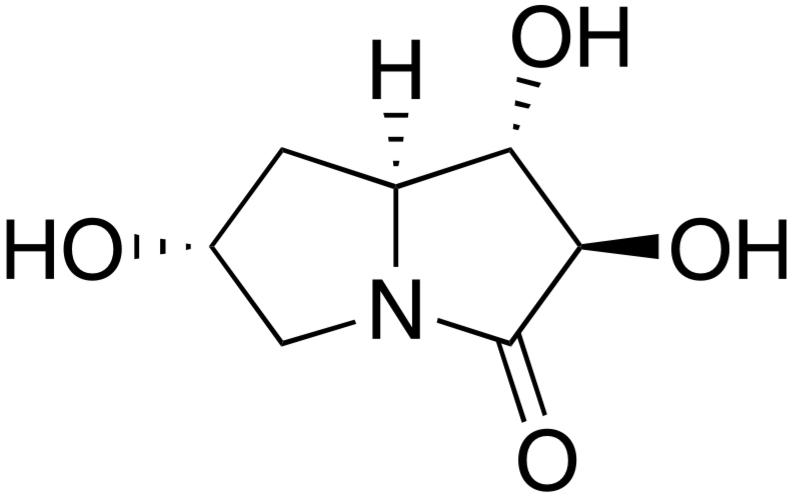


## Experimental

### 

#### Crystal data

C_7_H_11_NO_4_
*M* *_r_* = 173.17
Monoclinic, 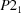

*a* = 4.6983 (3) Å
*b* = 14.5424 (10) Å
*c* = 5.5271 (4) Å
β = 99.663 (3)°
*V* = 372.28 (4) Å^3^
*Z* = 2
Cu *K*α radiation
μ = 1.09 mm^−1^
*T* = 100 K
0.31 × 0.27 × 0.25 mm

#### Data collection

Bruker Kappa APEXII DUO diffractometer
3697 measured reflections
1229 independent reflections
1228 reflections with *I* > 2σ(*I*)
*R*_int_ = 0.027

#### Refinement

*R*[*F* ^2^ > 2σ(*F* ^2^)] = 0.029
*wR*(*F* ^2^) = 0.073
*S* = 1.14
1229 reflections
112 parameters
1 restraint
H-atom parameters constrained
Δρ_max_ = 0.27 e Å^−3^
Δρ_min_ = −0.41 e Å^−3^
Absolute structure: Flack (1983 ▶), 537 Friedel pairs
Flack parameter: 0.20 (17)

### 

Data collection: *APEX2* (Bruker, 2010) ▶; cell refinement: *SAINT* (Bruker, 2010 ▶); data reduction: *SAINT*; program(s) used to solve structure: *SHELXS97* (Sheldrick, 2008 ▶); program(s) used to refine structure: *SHELXL97* (Sheldrick, 2008 ▶); molecular graphics: *WinGX* (Farrugia,1999 ▶) and *PLATON* (Spek, 2009 ▶); software used to prepare material for publication: *publCIF* (Westrip, 2010 ▶) and *PLATON*.

## Supplementary Material

Crystal structure: contains datablock(s) I, global. DOI: 10.1107/S1600536812002292/hb6566sup1.cif

Structure factors: contains datablock(s) I. DOI: 10.1107/S1600536812002292/hb6566Isup2.hkl

Supplementary material file. DOI: 10.1107/S1600536812002292/hb6566Isup3.cml

Additional supplementary materials:  crystallographic information; 3D view; checkCIF report

## Figures and Tables

**Table 1:** Hydrogen-bond geometry (Å, °)

*D*—H⋯*A*	*D*—H	H⋯*A*	*D*⋯*A*	*D*—H⋯*A*
O1—H1⋯O2^i^	0.84	1.98	2.8190 (15)	174
O2—H2⋯O1^ii^	0.84	2.50	3.1745 (15)	138
O2—H2⋯O4^iii^	0.84	2.25	2.8589 (15)	129
O4—H4⋯O3^iv^	0.84	1.84	2.6636 (15)	167
C4—H4*A*⋯O4^ii^	1.00	2.41	3.3057 (18)	148

Symmetry codes: (i) 
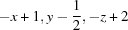
; (ii) 
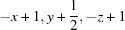
; (iii) 
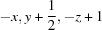
; (iv) 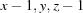.

**Fig. 1.**
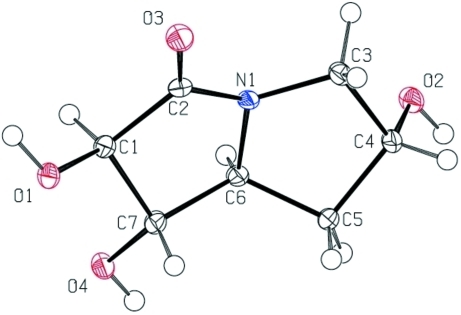
Molecular view of the title compound showing displacement ellipsoids drawn at the 50% probability level. H atoms are presented as a small spheres of arbitrary radius.

**Fig. 2.**
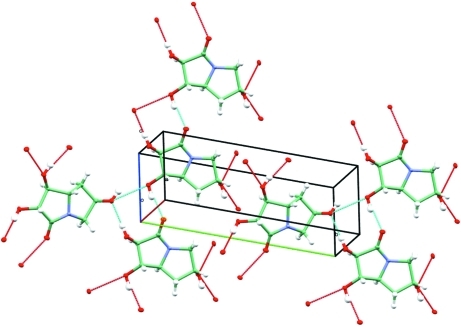
Title compound involved into hydrogen bonds. The presence of several hydroxyl groups in its structure leads this compound to behave as a sugar.

**Fig. 3.**
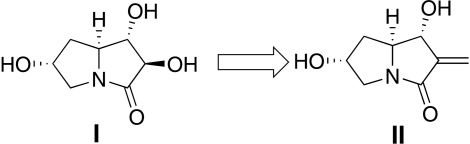
The conversion of (I) to pyrrolizidinone (II).

## supplementary crystallographic information

### Comment

Crystallographic data of the title polyhydroxylated pyrrolizidinone are disclosed. Compounds of this class can be used as glycosidase inhibitors and present a huge therapeutical potential for the treatment of a number of diseases such as cancer, diabetes, and lysosomal storage disorders (Baumann, 2007). The title compound has been prepared, for the first time, using a synthetic strategy based on a Morita-Baylis-Hillman adduct, easily obtained from a reaction between *N*-Boc-4(*R*)-hydroxy-2(*S*)-prolinal and methyl acrylate in 70% yield, as a mixture of diastereoisomers. After chromatographic separation, the minor isomer was transformed into the title compound. This compound was synthesized in five steps and 5.2% overall yield.

The asymmetric pyrrolizidinone, C_7_H_11_NO_4_, a new molecule with four stereocenters from a Morita-Baylis-Hillman adduct is shown in Fig. 1. The crystal packing (Fig. 2) is stabilized by hydrogen bonds. The dihedral angles of H7—C7—C6—H6 = 153.8° and H1A—C1—C7—H7 = 161.6° show that the H atoms 1A, 7 and 6 of the two new stereocenters are created in the *trans*-diaxial configuration. These values agree with the coupling constants values obtained for these protons in the ^1^H NMR analysis, ^3^J_H6,H7_ = 7.2 Hz e ^3^J_H1A,H7_ = 8.8 Hz. The crystallography parameters for this new molecule confirm its absolute configuration.

### Experimental

A solution of pyrrolizidinone (II) (0.10 g, 0.59 mmol) in MeOH/CH_2_Cl_2_ (3:7, 15 mL) was cooled to -72°C. After that a stream of oxygen/ozone was bubbled into it for 8–10 min (the reaction evolution was followed by TLC). Then, NaBH_4_ (0.112 g, 4.45 mmol) was added at -72°C and the resulting mixture was stirred for 6 h at room temperature. The reaction medium was initially acidified to pH 2–3 with a solution of HCl in methanol, then it was neutralized to pH 6–7 with solid Na_2_CO_3_. The resulting mixture was filtered over a pad of Celite^(^*^R^*^)^ and the solid was washed with methanol. The filtrates were combined and the solvents were removed under reduced pressure. The residue was purified by flash silica gel column chromatography (CH_2_Cl_2_:MeOH 95:05) to afford pyrrolizidinone I (0.08 g), as a white solid, in 80% yield. The title compound was recrystallized by using the liquid-vapor saturation method. The compound was dissolved with ethanol and crystallized with a vapor pressure of a second less polar liquid (chloroform), in a closed camera, providing the slow formation of crystals. [α]_D_^20^ + 3 (c 1, MeOH); *M*. p. 150–152°C; IR (KBr, *v*_max_): 3499, 3374, 2993, 2910, 1681, 1446, 1362, 1327, 1262, 1129, 1111, 1014 cm^-1^; ^1^H NMR (400 MHz, D_2_O) δ 1.78 (ddd, J 13.4, 5.0, 4.9 Hz, 1H, H-5B); 2.28 (dd, J 13.4, 5.7 Hz, 1H, H-5 A); 3.10 (d, J 12.8 Hz, 1H, H-3B; 3.77 (dd, J 12.9, 4.9 Hz, 1H, H-3 A); 3.91 (m, 1H, H-6); 3.99 (dd, J_H7,H1A_ 8.8, J_H6,H7_ 7.2 Hz, 1H, H-7); 4.60 (d, J_H7,H1A_ 8.8 Hz, 1H, H-1 A); 4.70 (t, J 4.9 Hz, 1H, H-4 A); ^13^C NMR (62.5 MHz, MeOD) δ 40.7, 52.1, 63.0, 73.2, 80.1, 83.3, 174.0; HRMS (ESI-TOF) Calcd. for C_7_H_12_NO_4_ [*M* + H]^+^ 174.0766, Found 174.0754.

### Refinement

The calculated Flack parameter was F=0.20 (17) (Flack, 1983). Analysis of the absolute structure was also performed using likelihood methods (Hooft *et al.*, 2008) as implemented in *PLATON* (Spek, 2009). The resulting value for the Hooft parameter was *y*=0.12 (4), with a corresponding probability for an inverted structure smaller than 1 × 10^-100^. Taken togheter, these results indicate that the absolute structure has been determined correctly.

### Crystal data

**Table:**

C_7_H_11_NO_4_	*F*(000) = 184
*M**_r_* = 173.17	*D*_x_ = 1.545 Mg m^−^^3^
Monoclinic, *P*2_1_	Cu *K*α radiation, λ = 1.54178 Å
*a* = 4.6983 (3) Å	Cell parameters from 1229 reflections
*b* = 14.5424 (10) Å	θ = 6.1–66.8°
*c* = 5.5271 (4) Å	µ = 1.09 mm^−^^1^
β = 99.663 (3)°	*T* = 100 K
*V* = 372.28 (4) Å^3^	Rectangular block, colorless
*Z* = 2	0.31 × 0.27 × 0.25 mm

### Data collection

**Table:**

Bruker Kappa APEXII DUO diffractometer	1228 reflections with *I* > 2σ(*I*)
Radiation source: fine-focus sealed tube	*R*_int_ = 0.027
Graphite monochromator	θ_max_ = 66.8°, θ_min_ = 6.1°
Bruker APEX CCD area–detector scans	*h* = −5→5
3697 measured reflections	*k* = −16→16
1229 independent reflections	*l* = −6→6

### Refinement

**Table:**

Refinement on *F*^2^	Secondary atom site location: difference Fourier map
Least-squares matrix: full	Hydrogen site location: inferred from neighbouring sites
*R*[*F*^2^ > 2σ(*F*^2^)] = 0.029	H-atom parameters constrained
*wR*(*F*^2^) = 0.073	*w* = 1/[σ^2^(*F*_o_^2^) + (0.0498*P*)^2^ + 0.0546*P*] where *P* = (*F*_o_^2^ + 2*F*_c_^2^)/3
*S* = 1.14	(Δ/σ)_max_ = 0.012
1229 reflections	Δρ_max_ = 0.27 e Å^−^^3^
112 parameters	Δρ_min_ = −0.41 e Å^−^^3^
1 restraint	Absolute structure: Flack (1983), 537 Friedel pairs
Primary atom site location: structure-invariant direct methods	Flack parameter: 0.20 (17)

### Special details

**Table:**

Geometry. All e.s.d.'s (except the e.s.d. in the dihedral angle between two l.s. planes) are estimated using the full covariance matrix. The cell e.s.d.'s are taken into account individually in the estimation of e.s.d.'s in distances, angles and torsion angles; correlations between e.s.d.'s in cell parameters are only used when they are defined by crystal symmetry. An approximate (isotropic) treatment of cell e.s.d.'s is used for estimating e.s.d.'s involving l.s. planes.
Refinement. Refinement of *F*^2^ against ALL reflections. The weighted *R*-factor *wR* and goodness of fit *S* are based on *F*^2^, conventional *R*-factors *R* are based on *F*, with *F* set to zero for negative *F*^2^. The threshold expression of *F*^2^ > σ(*F*^2^) is used only for calculating *R*-factors(gt) *etc*. and is not relevant to the choice of reflections for refinement. *R*-factors based on *F*^2^ are statistically about twice as large as those based on *F*, and *R*- factors based on ALL data will be even larger.

### Fractional atomic coordinates and isotropic or equivalent isotropic displacement parameters (Å^2^)

**Table:**

	*x*	*y*	*z*	*U*_iso_*/*U*_eq_	
O1	0.6971 (2)	−0.03528 (7)	0.86162 (19)	0.0163 (3)	
H1	0.7222	−0.0594	1.0016	0.024*	
O2	0.2633 (2)	0.38620 (8)	0.6676 (2)	0.0167 (3)	
H2	0.1802	0.4123	0.5394	0.025*	
O3	0.9071 (2)	0.13035 (8)	1.12983 (19)	0.0194 (3)	
O4	0.1530 (2)	0.02523 (7)	0.5015 (2)	0.0167 (3)	
H4	0.0715	0.0504	0.3714	0.025*	
N1	0.5577 (3)	0.20182 (9)	0.8573 (2)	0.0128 (3)	
C1	0.5216 (3)	0.04341 (11)	0.8610 (3)	0.0134 (3)	
H1A	0.3599	0.0303	0.9528	0.016*	
C2	0.6890 (3)	0.12859 (11)	0.9697 (3)	0.0141 (3)	
C3	0.6663 (3)	0.29584 (11)	0.8577 (3)	0.0143 (3)	
H3A	0.8780	0.2966	0.8633	0.017*	
H3B	0.6191	0.3310	0.9992	0.017*	
C4	0.5078 (3)	0.33520 (10)	0.6142 (3)	0.0136 (3)	
H4A	0.6384	0.3753	0.5346	0.016*	
C5	0.4128 (3)	0.25050 (10)	0.4564 (3)	0.0142 (3)	
H5A	0.2409	0.2644	0.3318	0.017*	
H5B	0.5701	0.2286	0.3721	0.017*	
C6	0.3420 (3)	0.17927 (10)	0.6401 (2)	0.0124 (3)	
H6	0.1420	0.1887	0.6752	0.015*	
C7	0.3995 (3)	0.07635 (12)	0.6019 (3)	0.0132 (3)	
H7	0.5501	0.0697	0.4949	0.016*	

### Atomic displacement parameters (Å^2^)

**Table:**

	*U*^11^	*U*^22^	*U*^33^	*U*^12^	*U*^13^	*U*^23^
O1	0.0237 (5)	0.0097 (6)	0.0144 (5)	0.0043 (5)	0.0003 (4)	0.0025 (5)
O2	0.0219 (5)	0.0113 (6)	0.0152 (5)	0.0040 (4)	−0.0017 (4)	0.0004 (4)
O3	0.0229 (6)	0.0171 (6)	0.0149 (5)	0.0001 (5)	−0.0062 (4)	0.0016 (4)
O4	0.0209 (5)	0.0118 (6)	0.0141 (5)	−0.0016 (4)	−0.0065 (4)	0.0014 (4)
N1	0.0192 (6)	0.0101 (6)	0.0077 (6)	0.0004 (5)	−0.0019 (5)	−0.0002 (5)
C1	0.0175 (7)	0.0110 (7)	0.0111 (8)	0.0019 (6)	0.0007 (6)	0.0014 (5)
C2	0.0196 (7)	0.0140 (8)	0.0086 (7)	−0.0004 (6)	0.0021 (6)	−0.0007 (6)
C3	0.0172 (7)	0.0116 (8)	0.0130 (7)	−0.0010 (6)	−0.0009 (6)	−0.0009 (6)
C4	0.0185 (8)	0.0093 (7)	0.0124 (7)	0.0001 (6)	0.0007 (6)	0.0006 (5)
C5	0.0213 (7)	0.0106 (8)	0.0097 (7)	0.0001 (6)	−0.0005 (6)	0.0014 (6)
C6	0.0152 (7)	0.0113 (8)	0.0098 (7)	0.0010 (6)	−0.0005 (6)	0.0003 (6)
C7	0.0151 (7)	0.0120 (7)	0.0114 (7)	0.0002 (6)	−0.0007 (6)	0.0020 (6)

### Geometric parameters (Å, º)

**Table:**

O1—C1	1.410 (2)	C1—H1A	1.0000
O1—H1	0.8400	C3—C4	1.535 (2)
O2—C4	1.439 (2)	C3—H3A	0.9900
O2—H2	0.8400	C3—H3B	0.9900
O3—C2	1.2368 (19)	C4—C5	1.532 (2)
O4—C7	1.409 (2)	C4—H4A	1.0000
O4—H4	0.8400	C5—C6	1.526 (2)
N1—C2	1.331 (2)	C5—H5A	0.9900
N1—C3	1.459 (2)	C5—H5B	0.9900
N1—C6	1.4721 (18)	C6—C7	1.542 (2)
C1—C7	1.528 (2)	C6—H6	1.0000
C1—C2	1.535 (2)	C7—H7	1.0000
			
C1—O1—H1	109.5	C5—C4—C3	104.56 (12)
C4—O2—H2	109.5	O2—C4—H4A	111.1
C7—O4—H4	109.5	C5—C4—H4A	111.1
C2—N1—C3	127.91 (12)	C3—C4—H4A	111.1
C2—N1—C6	113.83 (12)	C6—C5—C4	104.01 (12)
C3—N1—C6	113.74 (12)	C6—C5—H5A	111.0
O1—C1—C7	112.59 (13)	C4—C5—H5A	111.0
O1—C1—C2	113.15 (12)	C6—C5—H5B	111.0
C7—C1—C2	101.60 (12)	C4—C5—H5B	111.0
O1—C1—H1A	109.7	H5A—C5—H5B	109.0
C7—C1—H1A	109.7	N1—C6—C5	101.20 (12)
C2—C1—H1A	109.7	N1—C6—C7	102.44 (12)
O3—C2—N1	125.51 (15)	C5—C6—C7	120.32 (12)
O3—C2—C1	127.26 (14)	N1—C6—H6	110.6
N1—C2—C1	107.22 (11)	C5—C6—H6	110.6
N1—C3—C4	103.30 (12)	C7—C6—H6	110.6
N1—C3—H3A	111.1	O4—C7—C1	110.98 (13)
C4—C3—H3A	111.1	O4—C7—C6	114.49 (13)
N1—C3—H3B	111.1	C1—C7—C6	102.87 (12)
C4—C3—H3B	111.1	O4—C7—H7	109.4
H3A—C3—H3B	109.1	C1—C7—H7	109.4
O2—C4—C5	111.40 (12)	C6—C7—H7	109.4
O2—C4—C3	107.38 (12)		

### Hydrogen-bond geometry (Å, º)

**Table:**

*D*—H···*A*	*D*—H	H···*A*	*D*···*A*	*D*—H···*A*
O1—H1···O2^i^	0.84	1.98	2.8190 (15)	174
O2—H2···O1^ii^	0.84	2.50	3.1745 (15)	138
O2—H2···O4^iii^	0.84	2.25	2.8589 (15)	129
O4—H4···O3^iv^	0.84	1.84	2.6636 (15)	167
C4—H4*A*···O4^ii^	1.00	2.41	3.3057 (18)	148

Symmetry codes: (i) −*x*+1, *y*−1/2, −*z*+2; (ii) −*x*+1, *y*+1/2, −*z*+1; (iii) −*x*, *y*+1/2, −*z*+1; (iv) *x*−1, *y*, *z*−1.
